# The Influence of Headform Friction and Inertial Properties on Oblique Impact Helmet Testing

**DOI:** 10.1007/s10439-024-03460-w

**Published:** 2024-02-29

**Authors:** Nicole E.-P. Stark, Mark Begonia, Luca Viano, Steven Rowson

**Affiliations:** 1 Department of Biomedical Engineering and Mechanics, Virginia Tech, 120 Kelly Hall, 325 Stanger Street MC 0298, Blacksburg, VA 24061 USA; 2 Institute for Critical Technology and Applied Science, Virginia Tech, Blacksburg, USA; 3 KASK S.p.a. ad unico socio Chiuduno, Chiuduno, Italy

**Keywords:** Mass moment of inertia, Friction, Headforms, Oblique impacts, Impact kinematics

## Abstract

Helmet-testing headforms replicate the human head impact response, allowing the assessment of helmet protection and injury risk. However, the industry uses three different headforms with varying inertial and friction properties making study comparisons difficult because these headforms have different inertial and friction properties that may affect their impact response. This study aimed to quantify the influence of headform coefficient of friction (COF) and inertial properties on oblique impact response. The static COF of each headform condition (EN960, Hybrid III, NOCSAE, Hybrid III with a skull cap, NOCSAE with a skull cap) was measured against the helmet lining material used in a KASK prototype helmet. Each headform condition was tested with the same helmet model at two speeds (4.8 & 7.3 m/s) and two primary orientations (y-axis and x-axis rotation) with 5 repetitions, totaling 100 tests. The influence of impact location, inertial properties, and friction on linear and rotational impact kinematics was investigated using a MANOVA, and type II sums of squares were used to determine how much variance in dependent variables friction and inertia accounted for. Our results show significant differences in impact response between headforms, with rotational head kinematics being more sensitive to differences in inertial rather than frictional properties. However, at high-speed impacts, linear head kinematics are more affected by changes in frictional properties rather than inertial properties. Helmet testing protocols should consider differences between headforms’ inertial and frictional properties during interpretation. These results provide a framework for cross-comparative analysis between studies that use different headforms and headform modifiers.

## Introduction

**Fig. 1:**
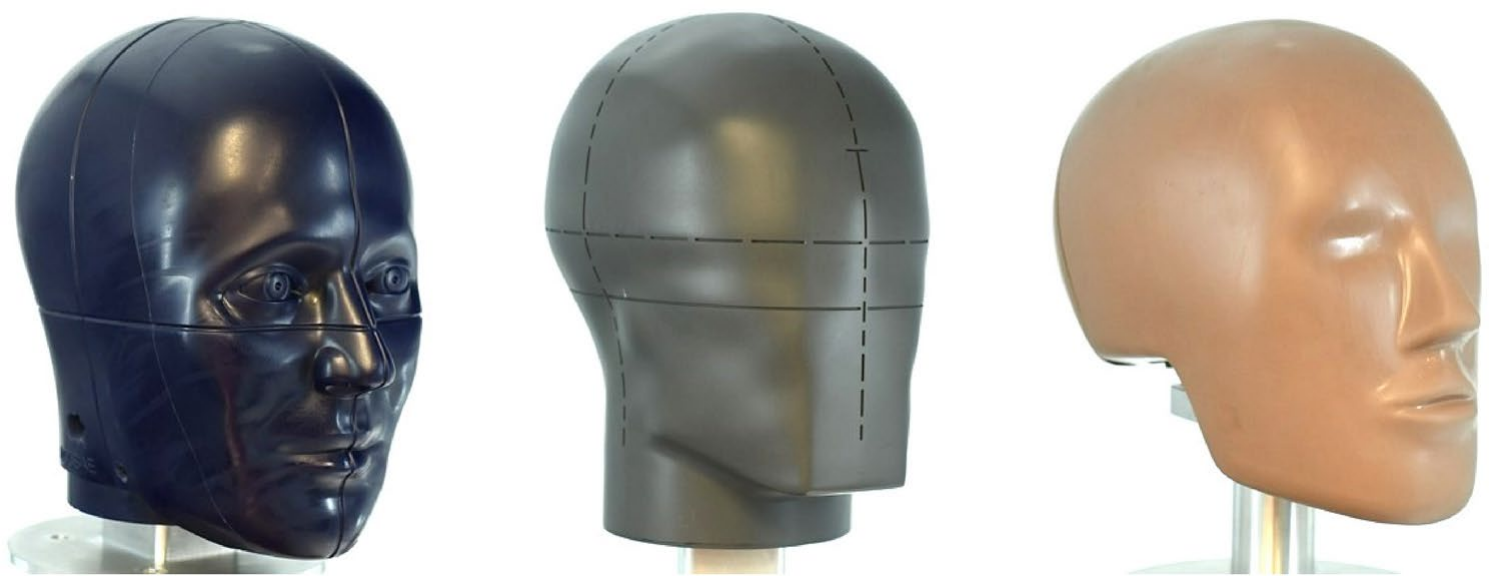
The NOCSAE (left), EN960 (middle), and Hybrid III (right) headforms. The NOCSAE headform has a polyurethane skin [17] and the Hybrid III has a vinyl plastisol skin. [18, 35] The EN960 is composed of magnesium

Helmet-testing headforms replicate human head impact response to assess helmet protection and injury risk. US and European product safety standards evaluate bicycle helmets for minimum protection standard against peak linear acceleration with a high risk of catastrophic head injury. These safety standards often use a linear drop tower where a helmeted headform sustains impacts that are normal to different anvils [11, 19]. However, real-world head impacts often occur in an oblique direction relative to the impact surface, causing both linear and rotational head impact kinematics [3, 25]. Therefore, assessing helmets solely based on linear acceleration is inadequate for fully understanding their overall protection against traumatic brain injuries. Thus, oblique impact testing is used to assess the helmet’s ability to reduce the rotational response of the head during impact, which is correlated to brain strain, and linear response, which is correlated to intracranial pressure gradients [16, 21, 28].  By incorporating oblique impact testing, a more comprehensive understanding of the helmet’s effectiveness in managing linear and rotational kinematics is achieved. There are three commonly used headforms for various standards, independent ratings, and research methods [1, 4, 5, 7, 8, 12, 14, 17, 26, 27, 33]. Included are the EN960 headform, a magnesium alloy headform that is frequently implemented in standards for helmet testing [12, 27, 33] the Hybrid III headform, initially developed for automotive crash testing [18, 35], and the National Operating Committee on Standards for Athletic Equipment (NOCSAE) headform, designed for sports helmet testing (Fig. 1) [7, 14, 17]. Comparing studies across the industry becomes notably challenging due to the use of three different headforms, each possessing distinct inertial and frictional properties that could alter their impact responses.

These three headforms have distinct outer material compositions, resulting in varying headform-helmet frictional interfaces that could influence impact kinematics [32]. Bonin et al. evaluated the effect of friction on impact kinematics by reducing the friction of the Hybrid III headform through the addition of a skull cap or a wig [6]. They found that this led to a reduction in rotational acceleration and no changes in linear acceleration [6]. Other studies have reported increased rotational acceleration with increased friction after adding a rubber layer to a magnesium headform [12, 27]. Moreover, modifying the friction of a singular headform has been observed to affect rotational, but not linear, kinematics when tangential speed is altered [27]. However, the effects of varying frictional properties across all three headforms have yet to be fully understood. Additional research is essential to ascertain if the observed effects on rotational and linear accelerations are consistent across various headforms.

Previous research found that the EN960 headform, NOCSAE headform with a skull cap, and Hybrid III headform with a skull cap have coefficients of friction (COFs) that are similar to the human head, while bare Hybrid III and NOCSAE headforms have significantly higher COFs, all tested at the same normal force of 80 N [32]. The COF of the human head has been found to be 0.30–0.34 against EPS foam and 0.21–0.35 against polyester [31, 33]. In another attempt to reduce the friction to the human head level, one study evaluated adding another slip plane (e.g., porcine scalp), finding a reduction in rotational acceleration [34]. However, it remains unclear if the decrease in rotational acceleration was from reduced friction or the increased energy absorption and sliding motion from the additional material [34]. Another recent study compared two headforms with different friction and inertial properties and found alterations in rotational impact response [36]. However, there was no specification if the response changes were from friction or inertial differences in the headforms.

Each headform also has distinct inertial properties that significantly affect oblique impact response [9, 10, 15, 24]. Compared to a human head with the same circumference, the EN960 has a higher mass, x-axis moment of inertia (MOI), and y-axis MOI. [9, 10] The Hybrid III has a more realistic mass and x-axis MOI but has a high y-axis and z-axis MOI compared to the human head [9, 10]. The NOCSAE headform has a similar mass to the Hybrid III, but higher x-axis and y-axis MOI while having the most human-like z-axis MOI [10, 13]. Research comparing headforms with varying MOI have found differences in impact rotational kinematics [10, 13]. However, these studies did not take into account the frictional differences between headforms (Fig. 1).

Evaluating the effects of both inertial and frictional properties on impact response is necessary to fully understand the implications of using different headforms. This study aimed to quantify the influence of headform COF and inertial properties on oblique impact response. Due to the grabbing interaction of a high COF material, we hypothesized that increased headform COF would increase peak rotational velocity (PRV) and peak rotational acceleration (PRA), but not affect peak linear acceleration (PLA). We also hypothesized that inertial properties would have a larger effect on PRV and PRA than COF. Assessing COF and inertial properties' effect on oblique linear and rotational kinematics allows for an accurate interpretation between headforms. This information will highlight the influence of friction and inertia properties on oblique impacts, enabling a framework for cross-comparative analysis between studies and helping determine the focus of headform generation in the future.

## Materials and Methods

### Headform Friction and MOI

Using a specially designed tribometer, the static COF of each headform was measured against the helmet lining material used in a KASK prototype helmet, using the same methods detailed by Stark et al. [32] The KASK prototype helmet resembles their KASK Protone Icon bike helmet with the internal lining made of CoolMax® polyester fabric, with no deliberate rotation-mitigating technology. Headforms included the 50th percentile male Hybrid III headform (Humanetics, Farmington Hills, MI), 50th percentile male NOCSAE headform (Southern Impact Research Center, Rockford, TN), EN960 size J full headform (Cadex Inc., Richelieu, Quebec), NOCSAE headform with a skull cap (NIKE PRO Skull Cap 2.0), and Hybrid III headform with a skull cap (NIKE PRO Skull Cap 2.0). The designed tribometer includes a frame to securely mount the headform, a sled with a pancake load cell and CoolMax® polyester mounted on top, and tension load applicator connected to the sled with a tension load cell. The sled also has a low friction sliding surface that is accounted for and subtracted out in the calculation of the headform friction. Each headform static COF was captured by first securely mounting the headform and then applying an 80 N normal force to the tested material on the sled. A tangential force was then incrementally increased, measured with an in-line tension load cell, until movement was generated, breaking the static COF. The data processing and static COF were calculated using the methods described in Stark et al. [32] Each headform COF was measured five times against CoolMax® polyester fabric. The EN960 was not tested with a skull cap as the bare EN960 headform already has a lower COF than the bare NOCSAE and Hybrid III headforms [32]. The mass and MOIs for each headform were defined using previously published data (Table 1) [10, 13, 20]. The predicted masses and MOIs for human heads, corresponding to the circumferences of the headforms, were derived from averaged data obtained from both a cadaver and CT study. [9, 10].

**Table 1:** Headform inertial properties obtained from averaged published data. [9, 10, 13, 20]

Headform	Circumference [mm]	Ixx [kg*cm^2^]	Iyy [kg*cm^2^]	Mass [kg]
NOCSAE	57.6	185.3	238.7	4.60
Hybrid III	59.0	159.6	224.4	4.54
EN960 J	57.5	244.0	312.5	4.70
Human	57.5	185.3	202.4	4.05
Human	59.0	211.3	228.2	4.40

### Oblique Impact Testing & Analysis

**Fig. 2:**
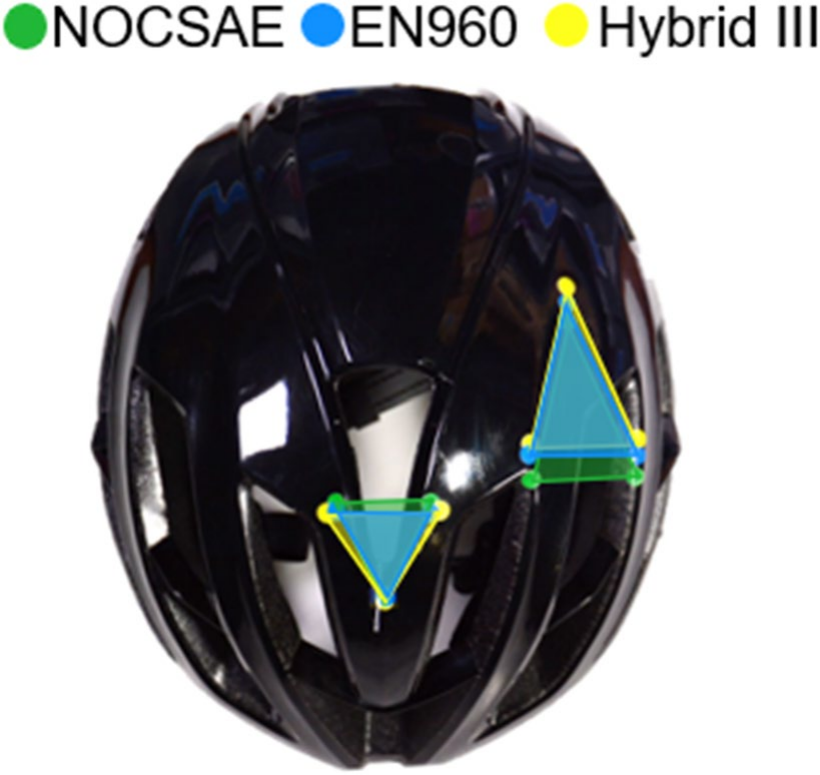
Comparison of impact locations for each headform (NOCSAE (green), EN960 (blue), Hybrid III (yellow)) and two primary orientations (y-axis and x-axis rotation, SAE J211 standard coordinate system). A custom 3-laser system was mounted onto the anvil to allow impact locations to be marked as each helmeted headform was positioned accordingly

**Fig. 3:**
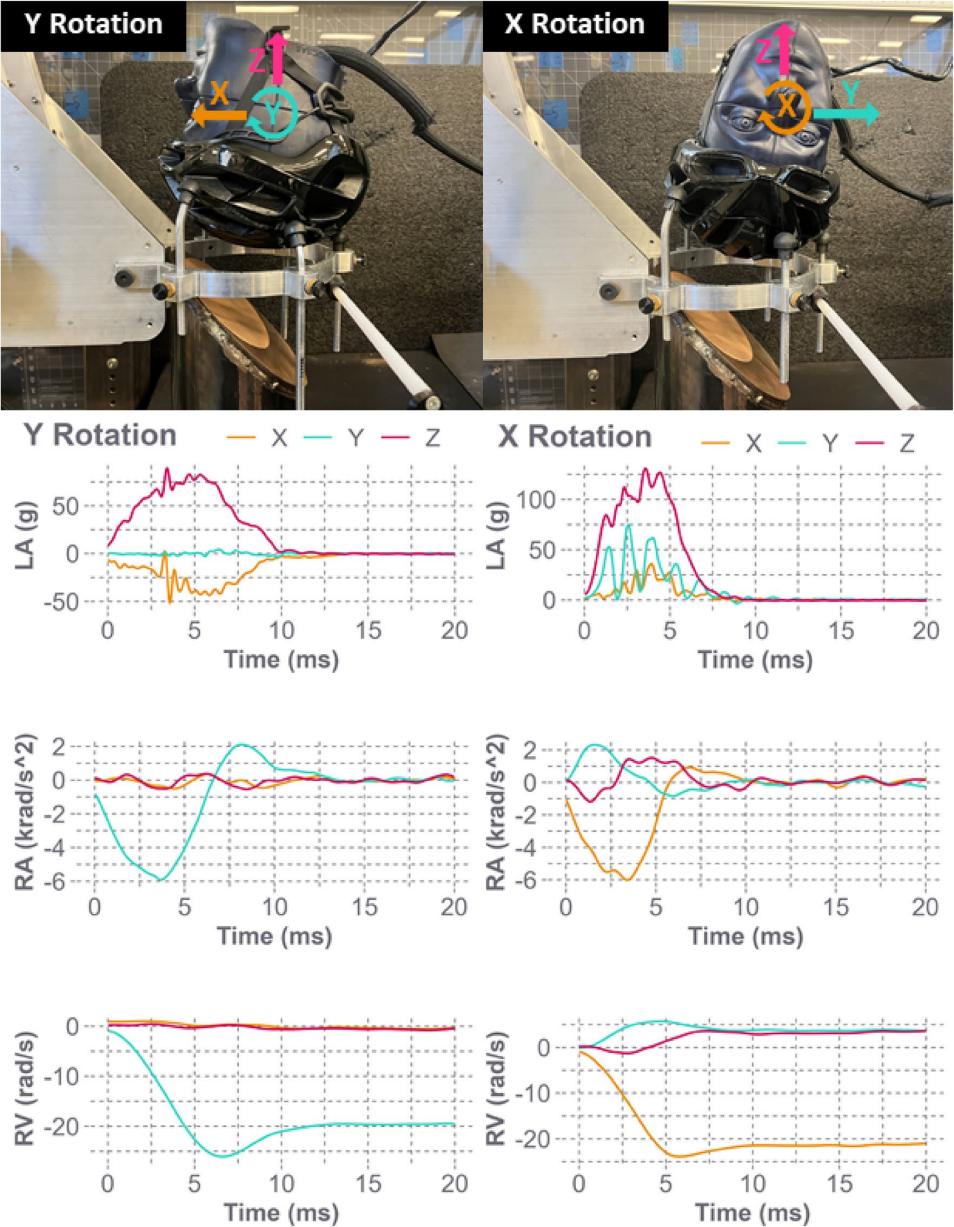
Oblique impact testing methods. Each helmeted headform was tested at two primary orientations (x-axis and y-axis rotation) at two speeds (4.8 and 7.3 m/s). Using a six-degree-of-freedom sensor package, three-axis linear acceleration (LA), rotational acceleration (RA), and rotational velocity (RV) data were collected. This is an example of x-axis and y-axis rotation impact for a NOCSAE 4.8 m/s impact and resulting LA, RA, and RV

Oblique impact testing was completed using guided drop tests of a helmeted headform (helmet, KASK prototype) onto a 45-degree anvil covered with 80-grit sandpaper. Each headform condition (EN960, Hybrid III, NOCSAE, Hybrid III with a skull cap, NOCSAE with a skull cap) was tested at two speeds (4.8 m/s: normal/tangential 3.39 m/s & 7.3 m/s: normal/tangential 5.16 m/s) and two primary orientations (y-axis and x-axis rotation, SAE J211 standard coordinate system (Fig. 2)) with 5 repetitions, totaling 100 tests. Each helmet was impacted twice, once for each primary orientation. Each of the 50 helmets was impacted at low speed first, then at high speed, and checked for no overlap of damage profiles. A dual-axis inclinometer, cross-level laser, and wall-mounted grid were used to ensure that the headforms were positioned consistently on the support ring of the drop tower. Each headform was instrumented with a six-degree-of-freedom sensor package, that consisted of three accelerometers (Endevco 7264B-2000, PCB Piezotronics, Depew, NY) and a tri-axis angular rate sensor (ARS) (ARS3 PRO, Diversified Technical Systems, Seal Beach, CA), mounted at the headform center of gravity. Data were collected at a sampling rate of 20 kHz and filtered using a 4-pole phaseless Butterworth low pass filter with a cutoff frequency of 1650 Hz for accelerometer signals (SAE J211) and 289 Hz for ARS signals (Fig. 3). Resultant PLA, PRA, and PRV were determined from each test.

The influence of primary orientation, inertial properties, and friction on PLA, PRV, and PRA was investigated using MANOVAs, significance level *α* < 0.05, in RStudio (Version 1.2, RStudio; Boston, Massachusetts, USA). The model used inertial and frictional properties, as well as primary orientation as independent variables, with PLA, PRA, and PRV as dependent variables. Type II sums of squares (SS) were used to determine how much variance friction and inertia accounted for in each dependent variable. The inertial properties depended on primary orientation and measure: mass for PLA, x-axis MOI (Ixx) for PRA and PRV for x-axis rotation impacts, and y-axis MOI (Iyy) for PRA and PRV for y-axis rotation impacts.

## Results

### Headform Friction and MOI

**Fig. 4:**
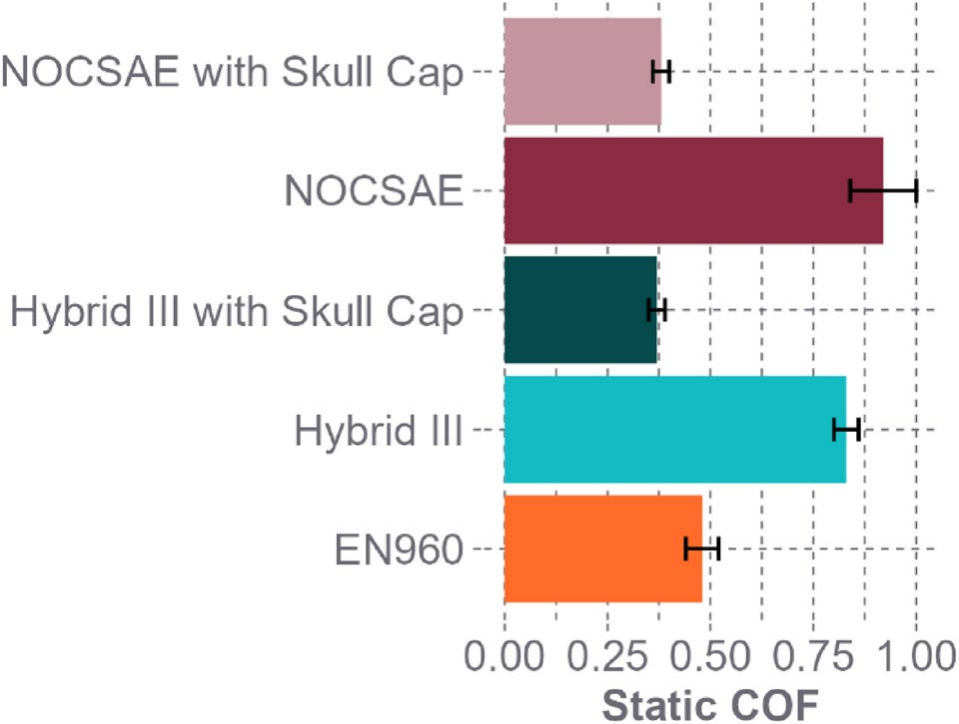
Coefficient of friction (COF) (mean ± standard deviation) for each headform. The NOCSAE and Hybrid III have high COFs that are reduced with a skull cap. The EN960 has a low COF

We quantified the COF of each headform against the helmet lining material (CoolMax® polyester fabric) used in a KASK prototype helmet (Fig. 4). The MOIs and masses for each headform were pulled from literature (Table 1). The EN960 had higher Ixx and Iyy than the other headforms, while the Hybrid III and NOCSAE had the highest COFs (Table 1). Though, with a skull cap applied, the NOCSAE and Hybrid III COFs decreased below the EN960.

### Oblique Impact Testing

In the y-axis primary orientation, all headform impact locations were less than 4 mm apart. In the x-axis primary orientation, all headform impact locations were less than 12 mm apart. Location contributed to a significant portion of the variance for high and low-speed impacts across PLA, PRA, and PRV (*p* < 0.01) (Tables 2 and 3).

**Table 2:** Low-speed (4.8 m/s) MANOVA sums of squares, percent of variance, and *p* values for each response measure

		PRV	PRA	PLA
COF	Sums of Squares	14	1.76*10^6^	514
	% Variance	1%*	2%	3%
	*p*	< 0.01	0.08	0.07
MOI	Sums of Squares	689	4.15*10^7^	76
	% Variance	53%*	48%*	0.5%
	*p*	< 0.01	< 0.01	0.48
Location	Sums of Squares	528	1.78*10^7^	7405
	% Variance	41%*	21%*	49%*
	*p*	< 0.01	< 0.01	< 0.01
Residuals	Sums of Squares	72	2.48*10^7^	7019
	% Variance	5%	29%	46%

The percent variance was calculated using type II SS (*p < 0.05)

**Table 3:** High-speed (7.3 m/s) MANOVA sums of squares, percent of variance, and *p* values for each response measure

		PRV	PRA	PLA
COF	Sums of Squares	48	1.08*10^7^	3253
	% Variance	2%*	4%*	32%*
	*p*	< 0.01	< 0.01	0.01
MOI	Sums of Squares	1332	1.48*10^8^	354
	% Variance	55%*	53%*	3%
	*p*	< 0.01	< 0.01	0.10
Location	Sums of Squares	891	8.29*10^7^	803
	% Variance	37%*	30%*	8%*
	*p*	< 0.01	< 0.01	< 0.01
Residuals	Sums of Squares	156	3.91*10^7^	5648
	% Variance	6%	13%	57%

The percent variance was calculated using type II SS (*p < 0.05)

**Fig. 5:**
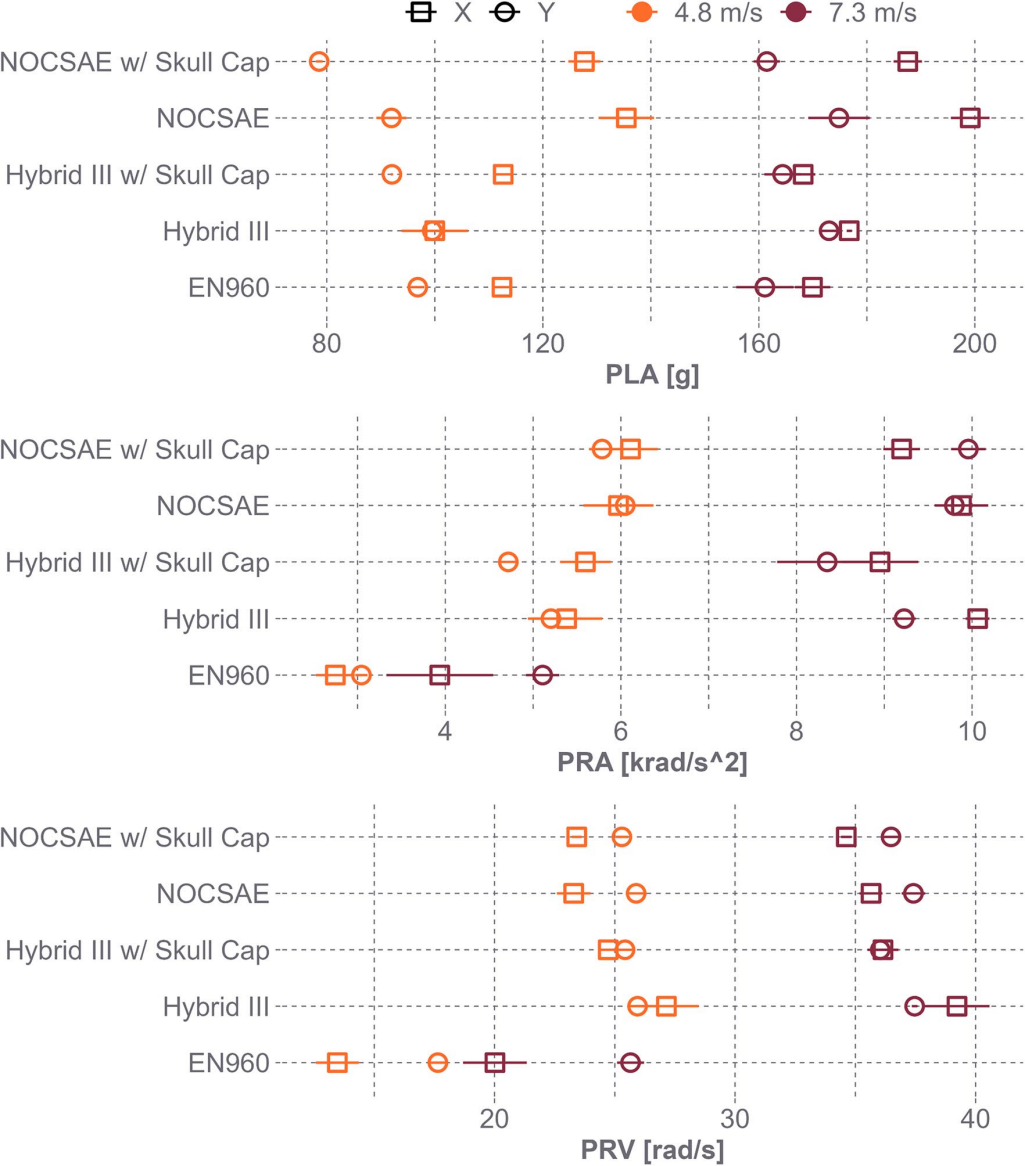
Summary of peak linear acceleration (PLA), peak rotational acceleration (PRA), and peak rotational velocity (PRV) for the five headform conditions at the two impact speeds (mean and 95% confidence intervals). The PLA decreased with changes in friction on a singular headform but was similar between the different headforms. The EN960 had lower PRA and PRV than the NOCSAE and Hybrid III

For the low-speed impacts, friction contributed to 3% of PLA variance (*p* = 0.07), 2% of PRA variance (*p* = 0.08), and 1% of PRV variance (*p* < 0.01) (Table 2). In comparison, inertia accounted for 0.5% of PLA variance (*p* = 0.48), 48% of PRA variance (*p* < 0.01), and 53% of PRV variance (*p* < 0.01). Notably, the variance in PRV attributable to friction, as well as the variance in PRA and PRV due to MOI was significant. In scenarios where a skull cap was added to headforms to reduce friction without altering MOI properties, there were slight decreases in PRV. Specifically, The NOCSAE PRV decreased 0.2 rad/s with a 0.87 reduction in friction, and the Hybrid III PRV decreased 2.9 rad/s with a 0.68 reduction in friction (Fig. 5). Larger decreases were found when comparing PRV across headforms with similar COF but different inertial properties: EN960 15.6 rad/s, Hybrid III with a skull cap 27.1 rad/s, and NOCSAE with a skull cap 24.4 rad/s. A similar trend was observed for PRA across headforms (Fig. 5).

For high-speed impacts (7.3 m/s), friction accounted for 32% of PLA’s variance (*p* < 0.01), 4% of PRA’s variance (*p* < 0.01), and 2% of PRV’s variance (*p* < 0.01) (Table 3). However, MOI accounted for more variance than friction, accounting for 53% of PRA’s variance (*p* < 0.01) and 55% of PRV’s (*p* < 0.01), but only 3% of PLA’s (*p* = 0.10). At high-speed impacts, both PRV and PRA had meaningful decreases due to friction, particularly when comparing bare headforms to those with a skull cap (Fig. 5).

Comparing different headform models, the EN960, which has a high MOI, had a lower PRV of 22.8 rad/s and PRA of 4.52 krad/s^2^. In contrast, the Hybrid III with a skull cap had a PRV of 36.1 rad/s and PRA of 8.65 krad/s^2^, while the NOCSAE with a skull cap had a PRV of 35.6 rad/s and PRA of 9.58 krad/s^2^ (Fig. 5). The addition of a skull cap to the NOCSAE headform demonstrated varying effects on different metrics when compared across location and speed. Specifically, the skull cap had a limited impact on PRV (*p* = 0.99) and PRA (*p* = 0.94), but it reduced PLA by 11.5 g (*p* = 0.04). Conversely, when a skull cap was added to the Hybrid III headform, there were notable decreases in all measures: PRV decreased by 2.4 rad/s (*p* < 0.01), PRA by 0.87 krad/s^2^ (*p* < 0.01), and PLA by 11.6 g (*p* = 0.04) (Fig. 5).

## Discussion

While previous studies have independently assessed the effects of friction and inertial properties of headforms on impact response, this study focuses on evaluating the combined influence of headform friction and inertial properties on oblique impact response across three commonly used headforms. Our findings suggest rotational impact kinematics are more sensitive to differences in inertial rather than frictional properties, though friction still significantly influenced rotational measures. We also observed that linear impact kinematics are more affected by frictional changes rather than differences in headform mass during high-speed impacts.

Our results show that MOI had the largest effect on rotational kinematics. This observation is particularly evident when comparing the NOCSAE and Hybrid III headforms, both fitted with skull caps, which exhibit similar COF and MOI. In contrast, the EN960 headform displays a distinctly different MOI. The addition of skull caps to the NOCSAE and Hybrid III headforms resulted in comparable friction levels, highlighting the unique contribution of the EN960's MOI to the observed variance in rotational kinematics. Previous research has shown that rotational kinematics is affected by friction [27]. Our results did show significant associations between COF and rotational kinematics, albeit much less than MOI. Altering the friction of a singular headform by adding a skull cap to the Hybrid III and NOCSAE resulted in slight changes in rotational impact kinematics. For high-speed impacts, reducing the friction of the NOCSAE headform from 0.92 to 0.38 accounted for a 4% decrease in PRA, a 2% decrease in PRV, and a 7% decrease in PLA. Similarly, adding a skull cap to the Hybrid III and that reduced the friction from 0.83 to 0.37 resulted in a 14% decrease in PRA, a 7% decrease in PRV, and a 12% decrease in PLA. This disparity in the magnitude of PLA, PRV, and PRA changes between the two headforms, when subjected to friction modifications, suggests a possible distinct influence of the skull cap's interaction with the headforms. However, it is important to note that this specific aspect was not directly tested in this study.

The decreases in rotational kinematics with reductions in friction were smaller herein compared to the decreases reported from previous studies that altered the friction of a singular headform. Bonin et al. found that when adding two stocking layers to a Hybrid III headform, reducing friction from 1.06 to 0.28, the PRA decreased by 27.5% and PRV by 22.9% [6]. Comparatively, our results had smaller reductions, which could in part be due to the difference in frictional interface, multiple repetitions, a different helmet model, different helmet sizes and fit, and/or the use of higher grit sandpaper on the anvil.

Other studies have also reported significant increases in rotational acceleration when increasing the friction of the EN960 headform by adding a rubber layer [12, 27]. Ebrahimi et at. found that when the COF of an EN960 headform is increased from 0.23 to 0.81, there was a 10% increase in PLA and an 89% increase in PRA. Juste-Lorente et al. reported a similar PLA increase of about 10% from 135 to 149 g for front impacts and 156 to 172 g for side impacts when friction was increased. However, Juste-Lorente et al. found a larger increase in PRA with friction increases, from 2168 to 9106 rad/s^2^ for front impacts and 3330 to 9050 rad/s^2^ for side impacts. Although the friction of an EN960 headform was not altered in this study due to its similarity to human friction [32], the effect of altering friction of the Hybrid III and NOCSAE was evaluated. Our results showed a comparable change in linear acceleration, with friction accounting for 3% of the PLA change in response at low-speed impacts and 32% at high-speed impacts. Still, our study found notably smaller changes in rotational acceleration. In contrast with the present study, Ebrahimi et al. and Juste-Lorente et al. tested using a motorcycle helmet, which could change the coupling between the headform and helmet. Ebrahimi et al. also used a steeper anvil angle, and Juste-Lorente et al. used a higher impact speed of 8.0 m/s; both anvil angle and impact speed would alter the rotational and linear head impact response. It is also important to note that Juste-Lorente et al. adjusted both the anvil angle and impact speed, changing the impact location and changing the MOI placement in the impact.

Evaluating the PLA variance attributed to friction, we found that high-speed friction contributed to 32% of the PLA variance, while low-speed friction accounted for 3%. This discrepancy may be due to the higher normal force, a potential increase in contact area, a larger crush area resulting from high-speed impacts, or a combination of these factors. However, our study did not explicitly evaluate these factors, and further research would be needed to examine them in detail.

Studies have also reported a significant reduction in rotational acceleration by adding a slip plane, such as a porcine scalp, to reduce headform COF [6, 34]. Our findings did not show a considerable reduction in rotational kinematics, accounting for only 1–4% of the variance in PRA and PRV. Adding a scalp could increase energy absorption, which may be the primary cause of the observed reduction in rotational kinematics and increased impact duration [6, 34]. Future studies should investigate the effects of adding a scalp on COF and energy absorption and how they contribute to impact kinematics.

Comparing headforms, a recent study found significant changes in PRA and PRV but similar PLA between two headforms with different friction and inertial properties [36]. However, this study did not look at the alteration in friction on the same headform with the same inertial properties, which prevented a comprehensive understanding of how friction affects impact response. While Yu et al. attributed the changes in rotational kinematics between headforms to friction and MOI, our findings indicate that these rotational kinematic changes might be more sensitive to MOI.

The headform with the largest x-axis and y-axis MOI, the EN960, produced a significantly lower rotational impact response compared to the Hybrid III and NOCSAE headforms. In contrast, the Hybrid III and NOCSAE headforms have similar MOI and thus exhibited similar rotational kinematics. Connor et al. compared the Hybrid III to the EN960, and while our findings showed a similar trend, the differences in rotational response between the headforms were 4.5 times larger in our study [10]. This could be attributed to the fact that we adjusted the impact location per headform to obtain a primary axis of rotation response. However, this was a minimal adjustment under 12 mm. At the same time, other studies align the helmets to the same orientation regardless of headform model. If we had impacted them all at the same location, MOI would have had confounding effects on the results because it would not be the exact axis of rotation.

There are several limitations to this study. First, only one helmet model was tested, and the results were unique to that helmet. Future testing with multiple helmet models or helmet models identical to those used in previous studies could help further isolate the effects of friction versus inertia. It is worth noting that the COF could vary for each headform [32], depending on the helmet lining material and the headform's age of use. Another limitation is that we did not test the EN960 with a skull cap, as this headform already has a lower COF, and skull caps are not commonly used on these headforms in literature [32]. Second, we only evaluated headform COF and MOI effects under oblique impact testing conditions. Trends in impact responses may not be the same for different test setups, such as a pendulum or pneumatic ram, or alternative boundary conditions like the inclusion of an attached neck. Third, the present study prioritized generating impact responses with a primary axis of headform rotation following a front or side hit to the helmet. Although measures were taken to ensure that each headform was oriented in the same manner and all alterations in impact location were less than 12 mm.

This study demonstrates that MOI differences between headforms can substantially influence rotational kinematics and that linear kinematics are more affected by the COF differences between headforms. This finding is important because of the relationship between rotational impact response and injury prediction [4, 22, 23, 29, 30]. However, this study cannot recommend a singular headform since it does not directly compare the biofidelity of each headform’s impact response. Also, inertial properties are broad across the population, and COF of a headform changes over time and against materials [9, 10, 32]. Although linear and rotational head kinematics measured in this study were specific to the headforms and test scenarios selected, the overall influence of friction and inertia remains applicable to assessments of helmet performance and injury prediction. Therefore, we recommend helmet testing protocols consider headform inertial and frictional properties when interpreting and comparing results. Furthermore, there needs to be an emphasis on biomechanical inertial properties for next-generation headform development. These results provide a framework for cross-comparative analysis between studies that use different headforms and headform alterations and investigate the implications in injury prediction between headforms.

## Abbreviations

CI: Confidence Interval
COF: Coefficient of Friction
EPS: Expanded Polystyrene
NOCSAE: National Operating Committee on Standards for Athletic Equipment
